# SWM dataset: A large-scale annotated benchmark for *Sclerotinia sclerotiorum* object detection in white mold management

**DOI:** 10.1016/j.dib.2026.113187

**Published:** 2026-08-19

**Authors:** Rubens de Castro Pereira, Ricardo da Silva Torres, Justino José Dias Neto, Díbio Leandro Borges, Murillo Lobo, Helio Pedrini

**Affiliations:** a Institute of Computing, University of Campinas, Campinas, 13083-852, SP, Brazil; b EMBRAPA Rice and Beans, GO-462, km 12, Santo Antônio de Goiás, 75375-000, GO, Brazil; c Institute of Informatics, Federal University of Goiás, Goiânia, 74690-900, GO, Brazil; d University of Brasília, Department of Computer Science, Brasília, 70910-900, DF, Brazil; e Artificial Intelligence Group, Wageningen University and Research, Droevendaalsesteeg 1, Wageningen, 6708 PB, Netherlands

**Keywords:** Phytopathometry, Early disease detection, Deep learning, Object detection, YOLO annotation, Pascal VOC annotation

## Abstract

Computer vision enables automated, accurate plant disease scouting, especially for diseases with patchy field distributions and lifecycles that begin in the soil and evolve across soil and plants. This is the case for the soilborne fungal pathogen *Sclerotinia sclerotiorum*, the causal agent of white mold in over 400 hosts, such as common bean, soybean, cotton, sunflower, canola, and tobacco, threatening sustainable agriculture on all continents. There are image datasets focused on plant disease detection, including the Plant Disease Database (PDDB) and PlantDoc, which cover a large range of plant diseases. None of them considers real-field images of *S. sclerotiorum* and white mold for detection. The novel dataset fills this gap by providing images of common bean crops annotated by plant pathologists, covering three life cycle stages: dormant sclerotia, germinated apothecia, and white mold symptoms. The image set is structured into two datasets: the SWM Dataset and the R-SWM Dataset. The SWM Dataset consists of a preprocessed and class-balanced version, whereas the R-SWM Dataset preserves the original raw-field images. Both datasets provide comprehensive annotations in YOLO and Pascal VOC formats, facilitating their adoption across different object detection models. The datasets are split into training, validation, and test sets, enabling the development of deep learning-based detection approaches to improve detector performance, address related challenges, and advance research in computer vision and plant pathology.

Specifications Table

**Table:** 

Subject	Computer Sciences
Specific subject area	Agronomy, Phytopathology, Plant disease, Deep learning, Object detection.
Type of data	Image in JPG format, and annotations in text format.
Data collection	We collected images of common bean (*Phaseolus vulgaris* L.) crops using smartphones from various models, including Samsung A51, S22, and S23, and iPhone 11 and 13. Common bean crops were located under center-pivot sprinkler irrigation at different phenological stages. The image acquisition occurred on different sunny or partially cloudy days, from 9 a.m. to 11 a.m. and 1 p.m. to 5 p.m. during the 2023 autumn and winter cropping season. The process specified that the digital camera must be positioned horizontally at 40–50 cm above the target, on the soil surface, or on plants. All images were used to structure the dataset, including the ground-truth annotations by the plant pathologists.
Data source location	The images were collected in common bean crops located in the following municipalities of Goiás state, Brazilian Center-West region: Vianópolis, Cristalina, Ipameri, Santa Helena de Goiás, and Turvelândia.
Data accessibility	Repository name: Sclerotinia and White Mold (SWM) dataset Data identification number: 10.17632/jxvmr8rchx.1 Direct URL to data: https://data.mendeley.com/datasets/jxvmr8rchx/1
Related research article	This paper supports our research reported in [1], which investigated the ensemble methods of state-of-the-art object detectors for detecting S. sclerotiorum and white mold.

## 1 Value of the Data

• The fungal soilborne pathogen *Sclerotinia sclerotiorum* is the causal agent of white mold (synonym: *Sclerotinia stem rot*) disease, which attacks over 400 hosts, including high-value crops with significant economic and social value. Some relevant host plants are common beans, soybeans, rapeseed, cotton, sunflower, canola, tobacco, and many other broad-leaf plants [2,3].
• The images were captured under field conditions in commercial common bean (*Phaseolus vulgaris*) farms infested with *S. sclerotiorum* and exhibiting white mold epidemics. The images primarily contain the following lifecycle stages: mature sclerotia, apothecia, and white mold symptoms. The images captured in the field contain many other objects and artifacts, which pose challenges for object detection tasks.
• The images were annotated by plant pathologists using the bounding-box technique to localize accurately and label regions containing target objects.
• The image dataset enables the development of computer vision and deep learning models for detecting the fungus and disease.
• The image dataset enables training, validation, and testing multiple object detection models for integration into plant disease detection applications.
• Researchers can use the image dataset as a benchmark to evaluate and compare the object detection performance of different algorithms.
• The object detection models trained on the SWM Dataset can help monitor plants and crops across all lifecycle stages of the pathogen and the disease. This monitoring is relevant to agronomists and farmers, enabling faster decision-making to prevent or manage the disease at early stages.

## 2 Background

Early plant disease detection is crucial for farmers to rapidly decide when to take action on crops, reducing yield losses and pesticide use in fields, and promoting more sustainable cropping. Among relevant plant diseases, white mold attacks staple foods and high-value cash crops, including common beans, soybeans, cotton, sunflowers, and many other broad-leaf plants, with significant economic and social impacts [4,5].

Tracking the progress of white mold in the crops is an essential task for the farmers as it can reduce the yield losses varying from 20% to 30% [6] in Brazilian soybean crops. The yield penalties exceed 90% in common beans, especially in environments with mild temperatures, moist weather, and ineffective disease management [7,8]. For this task, monitoring the pathogen’s life cycle and the onset of white mold using imaging is a promising alternative that relies on automated methods. Many studies have been conducted for the detection of crop diseases using images, often based on deep learning models [[9], [10], [11], [12], [13]] with encouraging results.

Many researchers aim to structure image datasets for plant disease detection, including Plant Disease Database (PDDB) [14], Plant Village [15], Plant Pathology Challenge 2020 [16], and PlantDoc [17]. To the best of our knowledge, none of them considers detecting *S. sclerotiorum* fungus and white mold from real-field images of common bean crops. We published original research in [1] and [18] that explores this new image dataset and focuses on improving object detection performance using detector-ensemble approaches.

## 3 Data Description

The dataset comprises images of common bean crops under real-field conditions. These images show three life stages of *S. sclerotiorum*: dormant sclerotia in the soil, apothecia germinated from sclerotia on the soil surface, and white mold symptoms on aerial plant parts, including leaves, stems, and pods.

In this dataset, each object class is assigned a numeric label ID or class name that links the bounding box annotations to the correct category. The label numbers and class names are: (1) Apothecium, (3) Mature Sclerotium, and (5) White Mold. These label numbers are not continuous meaning that the skipped numbers are reserved for future use. The dataset includes bounding box annotations in YOLO and Pascal VOC formats.

We organized the data into two datasets: the SWM Dataset and the R-SWM Dataset. The SWM Dataset was derived from the original R-SWM Dataset by cropping images to fixed dimensions, providing a standardized and class-balanced benchmark for training and evaluating object detection models under consistent experimental conditions. The experiments of the primary research used only the SWM Dataset [1,18]. The R-SWM Dataset preserves the original images at their native resolutions without preprocessing and was not used in the experiments reported in the primary research. It is available to support future studies on alternative preprocessing pipelines, high-resolution object detection, and other computer vision tasks that may benefit from the original image characteristics.

### 3.1 SWM dataset

The SWM Dataset comprises 2527 annotated images with dimensions (width and height) of 300×300 pixels, a resolution of 96 dpi, a bit depth of 24, and an RGB color space. This dataset is balanced across the classes. All original images were cropped to the same size using the bounding box annotations. Each annotated bounding box produced a new cropped image of 300×300 pixels, as this size is adequate for the evaluated object detection models in the primary research [1]. The bounding boxes were positioned at the center of the images, and the annotation data was adjusted accordingly. The target structures (mature sclerotia and apothecia) are sufficiently large in the original images, so cropping preserves their visual characteristics while ensuring uniformity. This standardized size facilitates fair comparison across detectors and aligns with common practice in object detection benchmarks. Table 1 presents the number of annotated objects per class across the training, validation, and test sets. The last row summarizes the number of annotations per set type and their percentage of the total.

**Table 1:** Number of annotated objects per class distributed in the training, validation, and test sets for the SWM Dataset. The last row shows the total number of annotations and the percentage per dataset type.

SWM Dataset								
Class	Training		Validation		Test		Total	
**Mature Sclerotium**	553	33.3%	186	34.1%	120	37.2%	859	34.0%
**Apothecium**	553	33.3%	173	31.7%	83	25.7%	809	32.0%
**White Mold**	553	33.3%	186	34.1%	120	37.2%	859	34.0%
**Total**	**1659**	**65.7%**	**545**	**21.6%**	**323**	**12.8%**	**2527**	**100.0%**

**Fig. 1:**
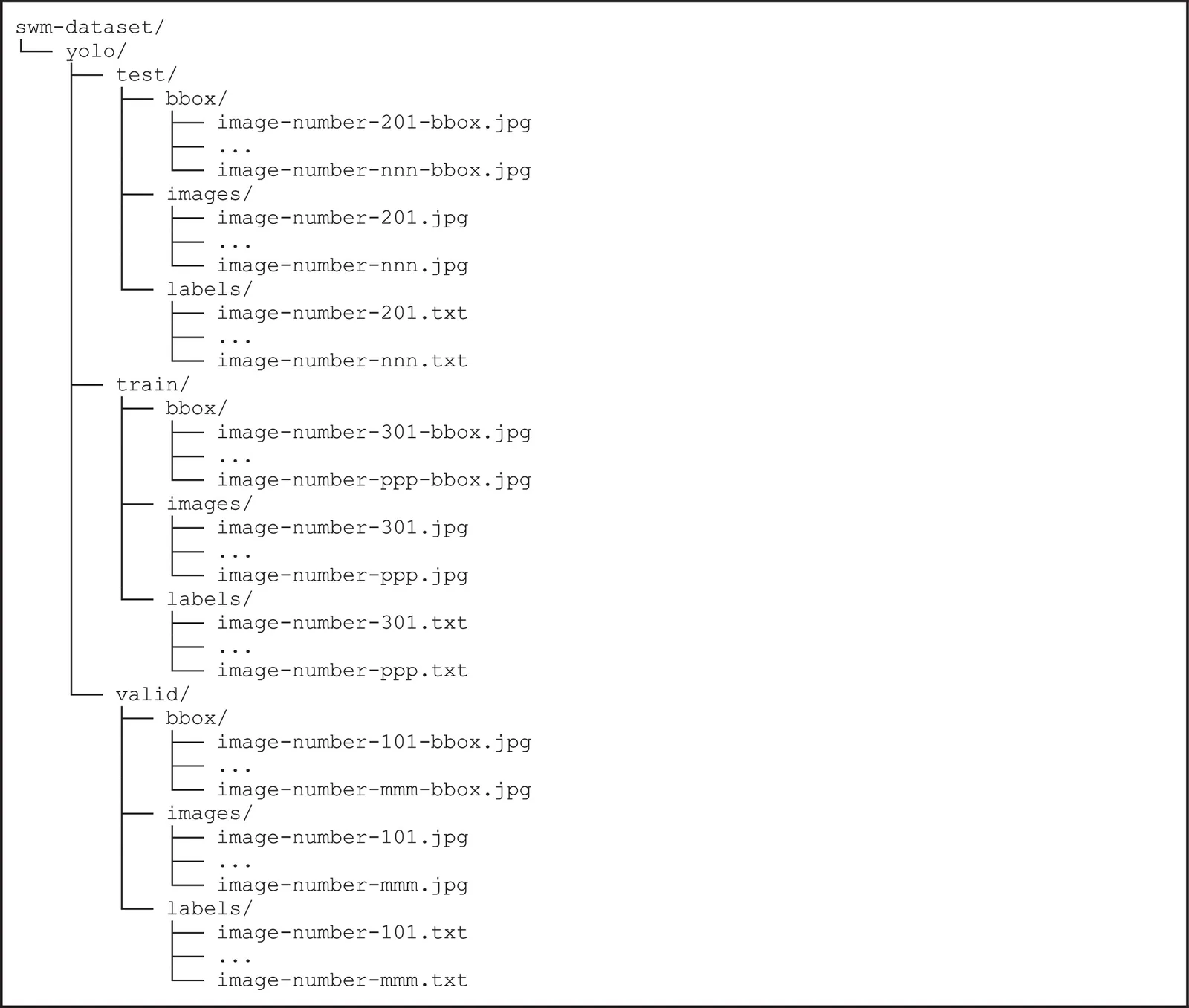
Folder structure for the SWM Dataset with YOLO annotations.

The SWM Dataset is divided into two annotation folders: “yolo” and “pascal_voc”. JSON and MS Excel files, containing details about the dataset, are also provided. Fig. 1 shows the folder structure for YOLO-formatted annotations. The “yolo” folder has the “test”, “train”, and “valid” folders. The next folder level comprises the “bbox” folder, which includes images with ground-truth bounding boxes, the “images” folder, which includes images in JPG (Joint Photographic Experts Group) format, and the “labels” folder, which contains the annotations file in YOLO format (one annotation file per image).

**Fig. 2:**
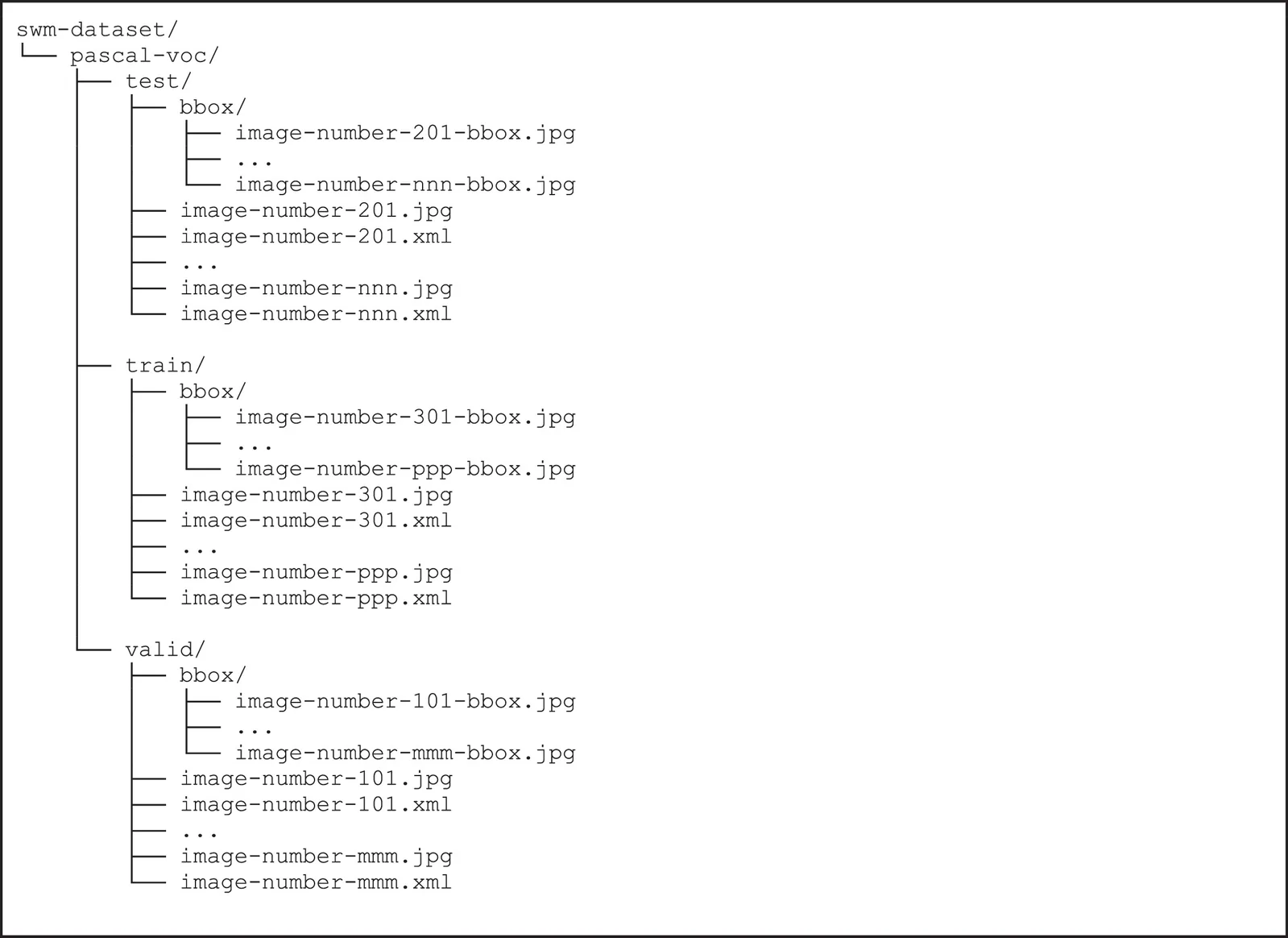
Folder structure for the SWM Dataset with Pascal VOC annotations.

Fig. 2 presents the folder structure for the annotations in Pascal VOC format. The “pascal-voc” folder has the “test”, “train”, and “valid” folders. The next folder level comprises the images in JPG format, the annotations file in the XML (Extensible Markup Language) format (one annotation file per image), and the “bbox” folder, which includes images with ground-truth bounding boxes.

**Fig. 3:**
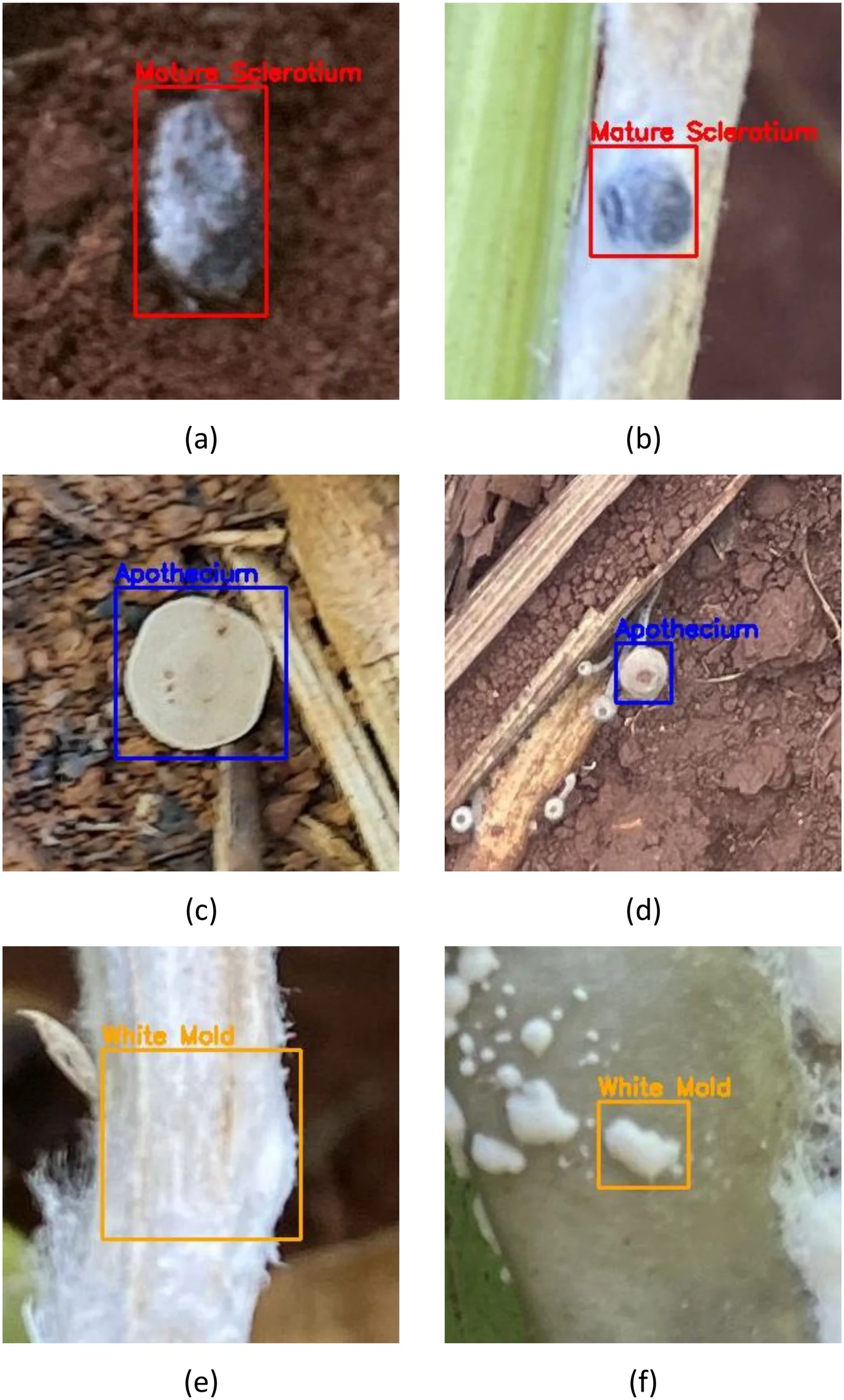
Examples from the SWM Dataset. (a)-(b) presents a mature sclerotium on the soil and on a pod. (c)-(d) are apothecia on the soil. (e)-(f) shows white mold on the stem and pod.

Fig. 3 shows image examples from the SWM Dataset with their ground-truth bounding boxes and class labels. (a)-(b) refer to the mature sclerotia on the soil and on a pod, (c)-(d) refer to apothecia on the soil, and (e)-(f) present the white mold on the stem and pod.

### 3.2 R-SWM dataset

The R-SWM Dataset consists of 1278 original (raw) images captured from the field with no preprocessing or filtering. The image dimensions ranged from 480 × 640 to 3024 × 4032 pixels, including resolutions of 1800 × 4000 and 3000 × 4000 pixels. All images were stored at 96 dpi resolution, with 24-bit depth and RGB color space.

Due to repository storage limitations, the folder structure differs slightly from that of the SWM Dataset. Specifically, we provide an “original-images” folder containing “train”, “test”, and “valid” subfolders, each of which includes “images” and “bbox” directories. To use the R-SWM Dataset for training and testing detector models, the images must be copied to the right folders according to the SWM Dataset structure presented in Fig. 1, Fig. 2.

**Fig. 4:**
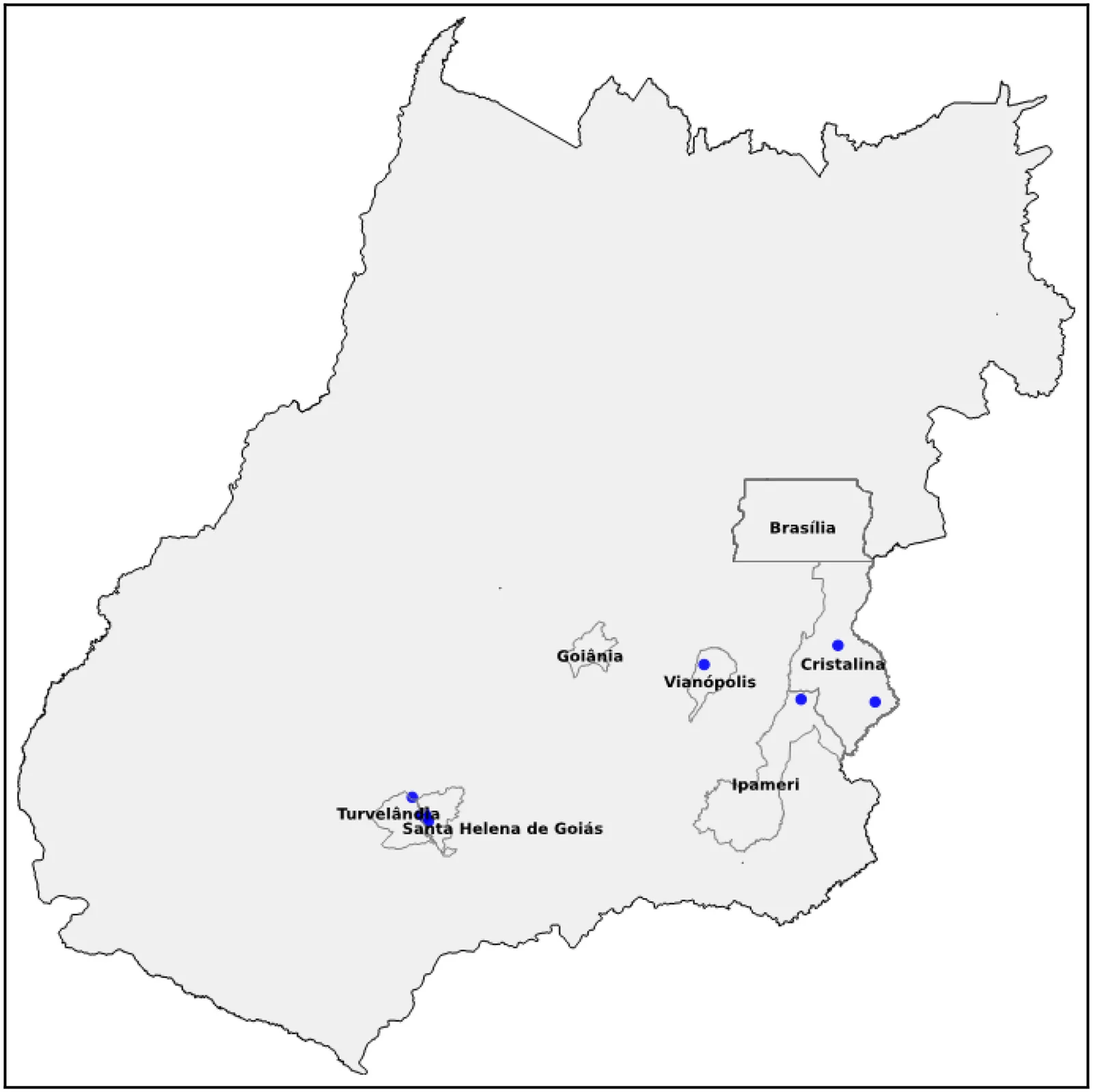
Map of Goiás State, Brazil, highlighting the areas where the images were collected in the blue points.

Table 2 presents detailed data on image capture, including the municipality, coordinates in decimal degrees, and the total number of images captured. Fig. 4 shows the distribution of the areas across the Brazilian state of Goiás, with blue points indicating the localization of the crops where the images were captured.

**Table 2:** Detailed localization of common bean crops at the farms with the total number of images captured.

Municipality and Brazil State	Localization	Number of Original Images
Vianópolis - Goiás	−16.7446880,−48.4811850	68
Cristalina - Goiás	−16.6156043,−47.5518928	50
Cristalina - Goiás	−16.9956271,−47.2938656	54
Ipameri - Goiás	−16.9780478,−47.8081116	48
Santa Helena de Goiás - Goiás	−17.7614690, −50.4258110	318
Santa Helena de Goiás - Goiás	−17.7695110, −50.4185030	61
Santa Helena de Goiás - Goiás	−17.7983060, −50.3967170	85
Santa Helena de Goiás - Goiás	−17.6359670, −50.5082670	133
Turvelandia - Goiás	−17.7486950, −50.4196670	62
Turvelandia - Goiás	−17.7616190, −50.4258190	43
Turvelandia - Goiás	−17.7506610, −50.4411610	150
Turvelandia - Goiás	−17.7660610, −50.4147470	37
Turvelandia - Goiás	−17.7662468, −50.4169464	148
Turvelandia - Goiás	−17.7509232, −50.4421425	21
**Total**		**1278**

Table 3 presents the total number of original images and the percentages allocated to the training, validation, and test sets for the R-SWM Dataset. Table 4 presents the number of annotations per class across the training, validation, and test sets. The last row summarizes the totals for annotations and the percentages for each dataset type, across all annotations.

**Table 3:** Number of images and percentage distributed in training, validation, and test set for the R-SWM Dataset.

R-SWM Dataset – Original Images								
	Training		Validation		Test		Total	
**Images**	894	70%	255	20%	129	10%	1278	100%

**Table 4:** Number of annotations per class distributed in the training, validation, and test sets for the R-SWM Dataset. The last row shows the total number of annotations and the percentage per dataset type.

R-SWM Dataset – Annotations								
Class	Training		Validation		Test		Total	
**Mature Sclerotium**	3369	42.2%	1013	46.7%	411	41.2%	4793	43.0%
**Apothecium**	629	7.9%	130	6.0%	100	10.0%	859	7.7%
**White Mold**	3988	49.9%	1028	47.4%	487	48.8%	5503	49.3%
**Total**	**7986**	**71.6%**	**2171**	**19.5%**	**998**	**8.9%**	**11,155**	**100.0%**

**Fig. 5:**
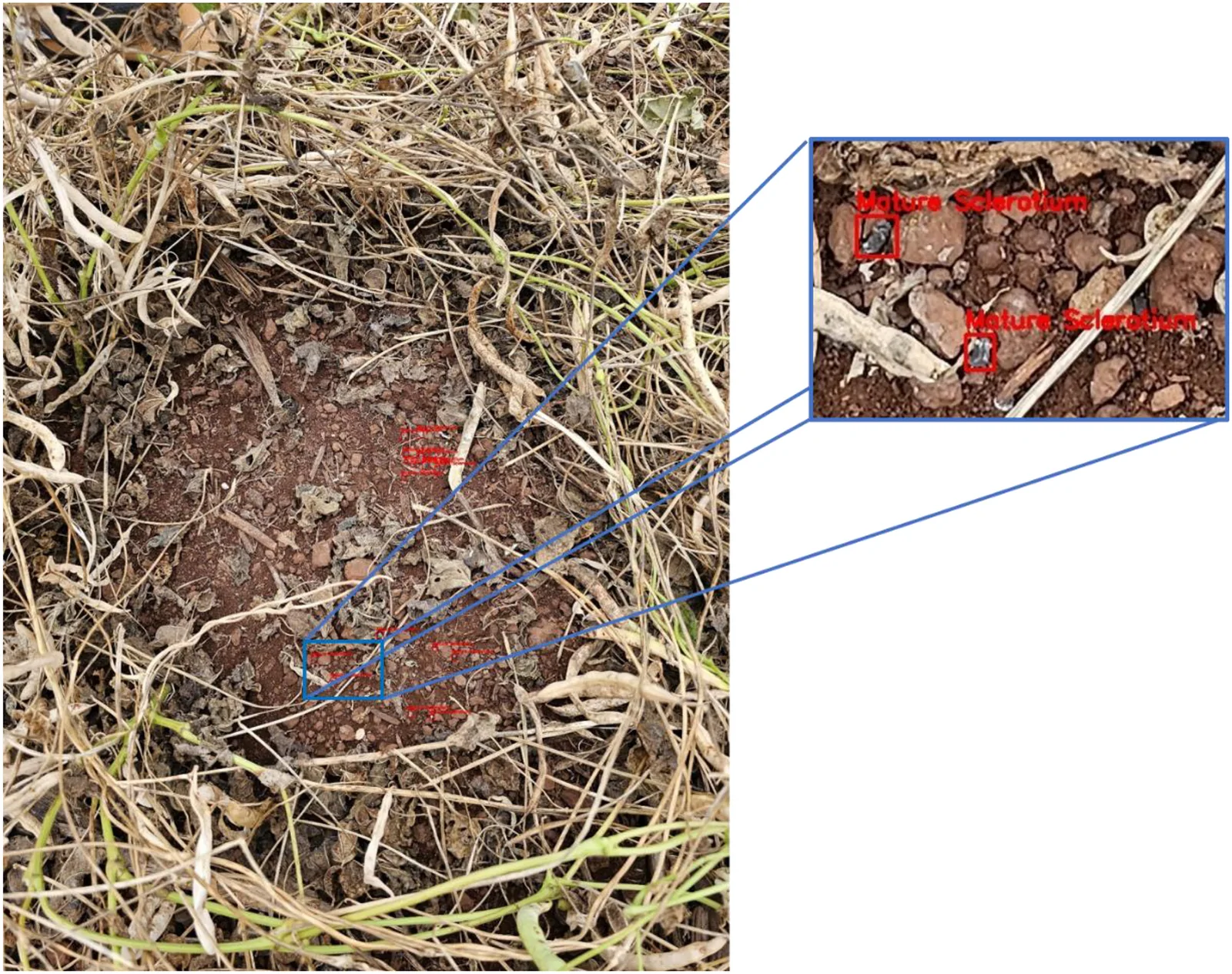
Original image from the R-SWM Dataset with mature sclerotia on the soil. The blue rectangle is zoomed at right to show the small structures of the mature sclerotia.

**Fig. 6:**
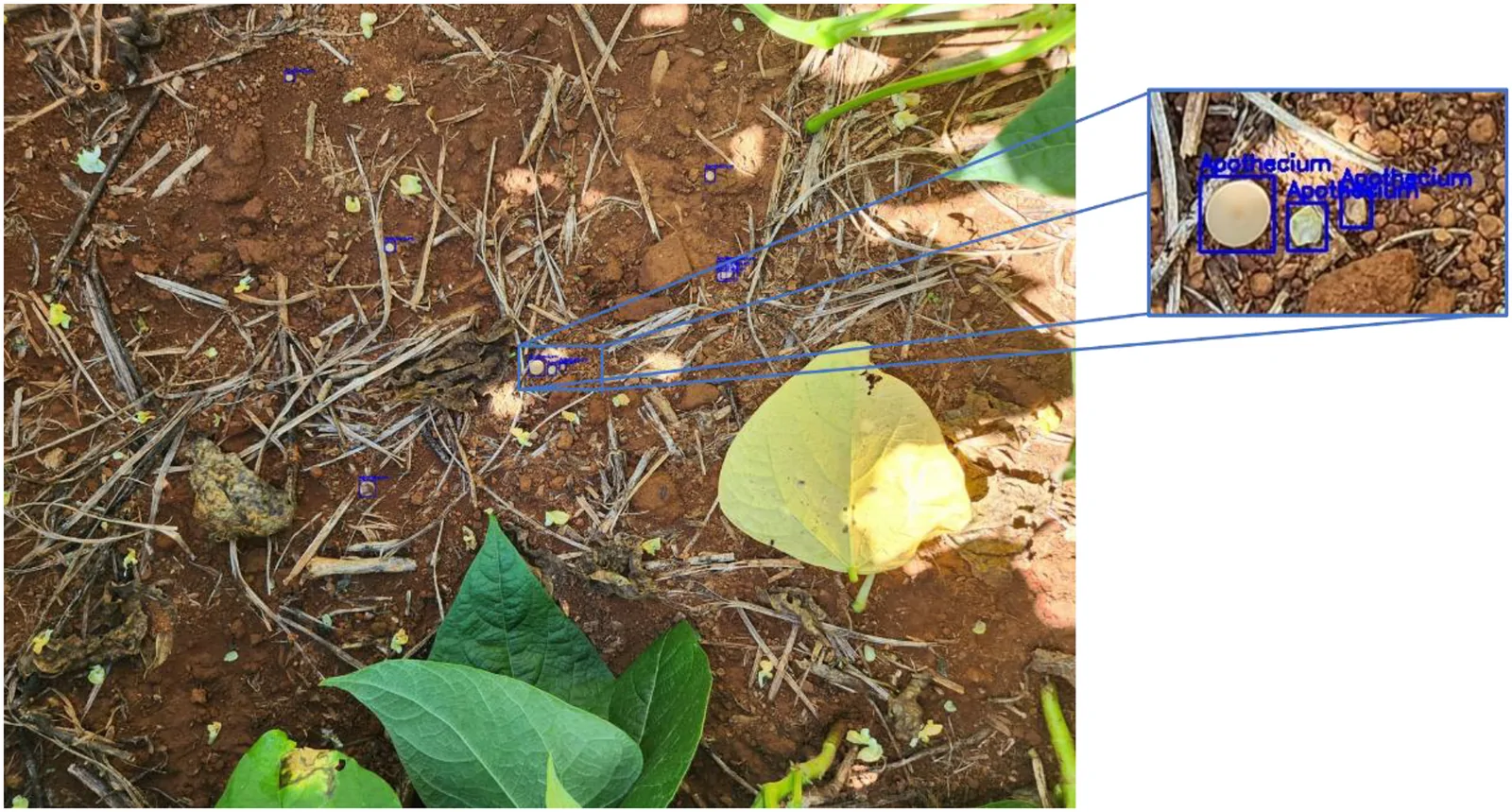
Example of original image from the R-SWM Dataset with apothecia on the soil. The right image is a zoom that highlights the apothecia structures and their morphological details.

**Fig. 7:**
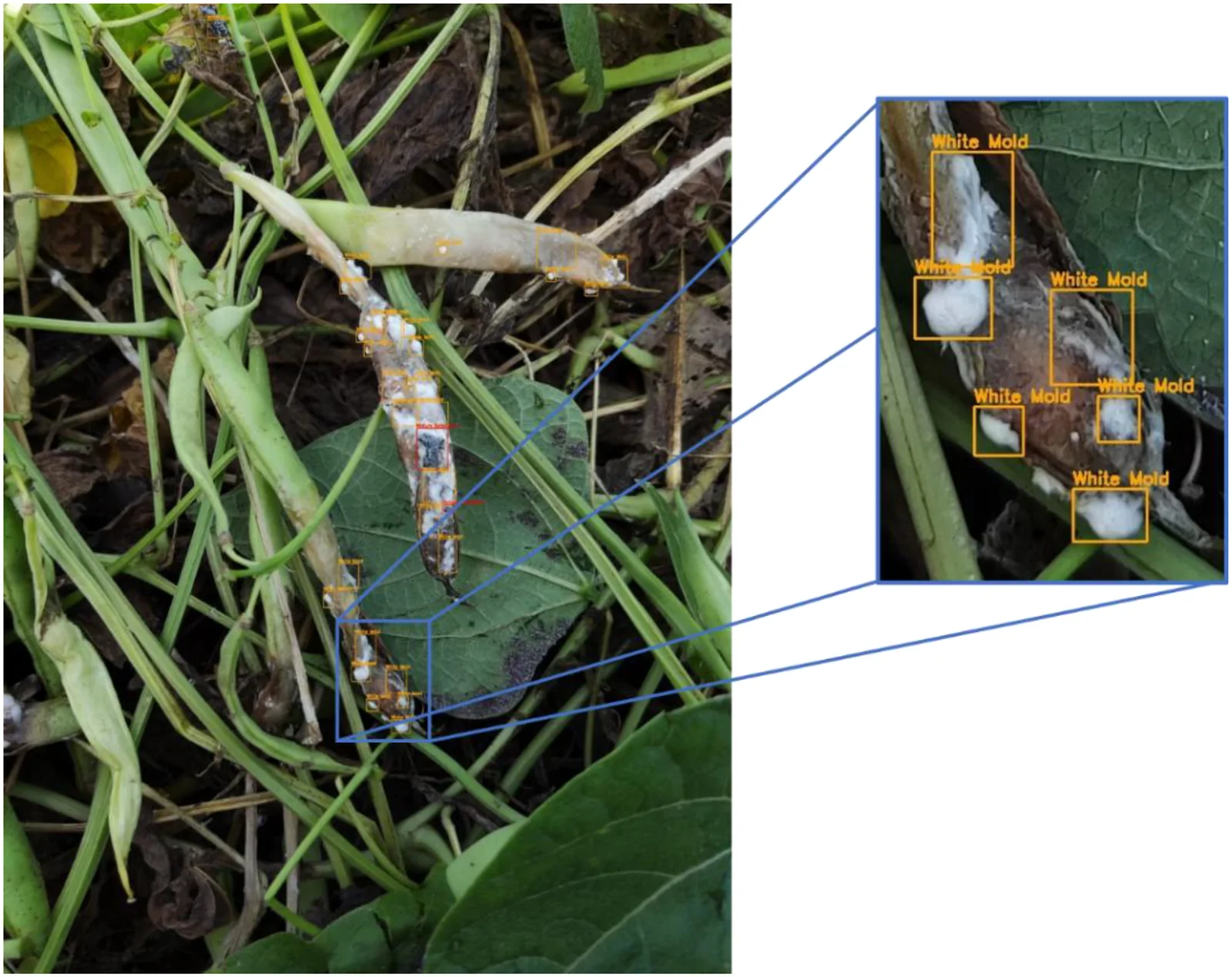
Original image from the R-SWM Dataset showing the white mold and newly formed sclerotia on a common bean plant. The right image is a zoom of the area highlighted by the rectangle, presenting white mold symptoms on a pod.

Fig. 5, Fig. 6, Fig. 7 present sample images from the R-SWM Dataset, along with ground-truth bounding boxes for the corresponding classes. Fig. 5 shows mature sclerotia in the soil, which appear small relative to the overall image. The right side shows a zoom-in from the blue box in the original image, highlighting two mature sclerotia near the stones, rocks, and other small objects. Fig. 6 shows apothecia in the soil and a zoom-in window highlighting three annotated apothecia. Fig. 7 shows pods infected with white mold, with six annotated areas by plant pathologists on the right.

### 3.3 YOLO format

The YOLO-format annotation files correspond to each annotated image. The file is a plain text file with no headers, where each row refers to one bounding box with the following values:

• Field 1: the label number related to the object class. The possible values are (1) Apothecium, (3) Mature Sclerotium, and (5) White Mold.
• Fields 2/3: the coordinates X (column) and Y (line) related to the central point of the bounding box. These values are normalized with respect to the image dimensions (width and height).
• Field 4: the bounding box width normalized with respect to the image width.
• Field 5: the bounding box height normalized with respect to the image height.

### 3.4 Pascal VOC format

The Pascal VOC format annotation files refer to each annotated image. Table 5 presents the XML tags related to the annotated bounding boxes.

**Table 5:** Description of annotation tags for Pascal VOC format.

Tag	Description	Type
<filename>	Image filename.	Text
<size> <width> … 〈/width〉 <height> … 〈/height〉 <depth> … 〈/depth〉 </size>	Image dimensions: width, height, and depth (channels).	Integer
<object> <name> … 〈/name〉 <pose>Unspecified</pose> <truncated>0</truncated> <difficult/> <bndbox> <xmin>1141</xmin> <ymin>2544</ymin> <xmax>1165</xmax> <ymax>2569</ymax> </bndbox> </object>	• Object: annotation data • name: class name – possible values: (Mature Sclerotium | Apothecium | White Mold) • pose: not used • difficult: not used • bndbox: bounding box data • xmin: the horizontal coordinate of the left edge of the box. • ymin: the vertical coordinate of the top edge of the box. • xmax: the horizontal coordinate of the right edge of the box. • ymax: the vertical coordinate of the bottom edge of the box.	Text Integer Integer Integer Integer

## 4 Experimental Design, Materials, and Methods

This section describes the systematic approach for building and validating the image dataset used in this research. The methodology is divided into two primary phases: i) the detailed technical aspects for image acquisition; and ii) protocols and tools used for expert annotations to produce the ground-truth bounding boxes.

### 4.1 Image acquisition

The images were acquired at commercial farms located in Vianópolis, Cristalina, Ipameri, Santa Helena de Goiás, and Turvelândia, all municipalities of the state of Goiás, in the Brazilian Center-West region. All these crops were grown under center pivot sprinkling irrigation, with mild temperatures and moist soil, conditions that are optimal for white mold epidemics. The common bean crops were at different phenological stages, from blooming to pod filling, illustrating the window of infection in most countries where white mold is important. Further, the typical fungal structures in soil and the disease symptoms in the image collection are found globally, ensuring the representativeness of the SWM Dataset. These images were collected by trained collaborators using smartphones of various models, including Samsung A51, S22, and S23, as well as iPhone 11 and 13. The images have variable dimensions around 4000×3000 pixels, a resolution of 96 dpi, a bit depth of 24, and RGB color.

Image acquisition took place on different sunny or partially cloudy days, from 9 a.m. to 11 a.m. and 1 p.m. to 5 p.m. during the 2023 autumn and winter cropping season. The image acquisition process involved positioning the digital camera horizontally at 40–50 cm from the target on the soil surface or on plants. Researchers sampled representative common bean fields with no other relevant biotic or abiotic stressors. The images depict the pathogen's developmental stages and the disease in different parts of the plant, including soil, leaves, stems, and pods.

### 4.2 Image annotations by experts

The plant pathologists annotated the images using the *Supervisely platform* [19], considering the two pathogen structures and the visible symptomatic stage, and labeled them in their respective classes: mature sclerotium, apothecium, and white mold. The mature sclerotia are visible on the soil surface or on lesions at advanced stages on the plant's upper parts, with varying epidemiological importance. The *S. sclerotiorum* life cycle is monocyclic, with no secondary infection cycles. Only older sclerotia formed in the previous season or in infected seeds that were sown germinate. Apothecia formation is the next stage after the older sclerotia germinate. This stage, known as carpogenic germination, normally occurs under a closed canopy during the blooming of host plants. The next stage occurs when the airborne ascospores reach the host flowers, initiating the disease onset. This symptomatic stage is named white mold. When there is enough moisture, the infection process is continuous with no clear interval between lesion expansion and the formation of new sclerotia [2,4]. Differentiating between white mold in active, expanding lesions and new sclerotia during formation is challenging because of their visual similarities.

The image background is typically noisy with many small objects, including small rocks on the soil, soil aggregates, straw, crop debris, coal pieces, saprophytic mushrooms, and dropped petals. Thus, all these elements in the image required considerable attention from specialists to produce high-quality annotations for the image dataset. This task produced an average of 8.7 annotations per original image. To ensure annotation quality, all annotations were independently reviewed by a second expert. Any discrepancies were discussed and resolved through consensus before the final ground-truth annotations were established. Since the annotation protocol was based on expert review and consensus rather than independent duplicate annotation of the entire dataset, quantitative inter-annotator agreement metrics (e.g., IoU or Cohen's kappa) were not applicable.

The object annotation used axis-aligned bounding boxes compatible with the YOLO and Pascal VOC formats. For approximately regular objects, such as mature sclerotia and apothecia, the bounding box was defined as the smallest rectangle enclosing the entire visible object. For irregular or diffuse white mold lesions on stems, leaves, and pods, the bounding box enclosed the entire visible symptomatic region, including all clearly identifiable diseased tissue while minimizing the inclusion of surrounding healthy tissue.

### 4.3 Dataset partitioning strategy

The SWM and R-SWM Datasets were randomly divided into training, validation, and test subsets at the image level, without stratification by field location or acquisition date. The dataset comprises images collected from six different municipalities captured across a broad range of times during the day. This random split strategy, combined with the heterogeneity of acquisition conditions, supports that the dataset is representative and considers diverse detection scenarios. Future evaluations may consider location- or time-based splits to better assess cross-field and cross-season robustness.

## Limitations

This section presents some limitations of our dataset as follows:

• The dataset does not consider other stages of the pathogen *S. sclerotiorum,* such as ascospore attachment on plant surfaces, infected flowers or the early, tiny and soaked lesions on the leaves, pods, or stems. Nevertheless, the dataset can be further expanded by incorporating additional images depicting other disease stages, without affecting the integrity or consistency of the current data.
• The dataset does not comprise segmentation masks for image segmentation, although this can be considered in future work using the images and the bounding boxes annotations.
• The dataset does not comprise any augmented data.
• The provided dataset split design may affect the generalization analysis of models.

## Ethics Statement

The authors have read and followed the ethical requirements for publication in Data in Brief and confirmed that the current work does not involve human subjects, animal experiments, or any data collected from social media platforms.

## Credit Author Statement

**Rubens de Castro Pereira**: Conceptualization, Methodology, Software, Validation, Formal Analysis, Investigation, Data Curation, Writing – original draft, Writing – review & editing, Visualization. **Ricardo da Silva Torres**: Conceptualization, Methodology, Validation, Formal Analysis, Investigation, Writing – review & editing. **Justino José Dias Neto**: Validation, Data Curation, Writing – review & editing, Visualization. **Díbio Leandro Borges**: Conceptualization, Methodology, Validation, Formal Analysis, Investigation, Writing – original draft, Writing – review & editing, Visualization. **Murillo Lobo Jr.**: Conceptualization, Methodology, Validation, Formal Analysis, Investigation, Resources, Funding, Data Curation, Writing – original draft, Writing – review & editing, Visualization. **Helio Pedrini**: Conceptualization, Methodology, Validation, Formal Analysis, Investigation, Writing – original draft, Writing – review & editing, Visualization.

## Data Availability

Mendeley DataSclerotinia and White Mold (SWM) dataset (Original data).
